# Acceptance and use of the Information System of the National Immunization Program*

**DOI:** 10.1590/1518-8345.3360.3307

**Published:** 2020-06-19

**Authors:** Valéria Conceição de Oliveira, Eliete Albano de Azevedo Guimarães, Gabriela Gonçalves Amaral, Talita Ingrid Magalhães Silva, Luciana Aparecida Fabriz, Ione Carvalho Pinto

**Affiliations:** 1 Universidade de São Paulo, Escola de Enfermagem de Ribeirão Preto, PAHO/WHO Collaborating Centre at the Nursing Development Research, Ribeirão Preto, SP, Brazil.; 2 Universidade Federal de São João del-Rei, Campus Centro Oeste Dona Lindu, Divinópolis, MG, Brazil.; 3 Scholarship holder at the Coordenação de Aperfeiçoamento de Pessoal de Nível Superior (CAPES), Brazil.; 4 Universidade Federal de Minas Gerais, Departamento de Enfermagem, Belo Horizonte, MG, Brazil.; 5 Scholarship holder at the Fundação Parque Tecnológico Itaipu/Fundação Araucária, Foz do Iguaçu, PR, Brazil.

**Keywords:** Health Information Systems, Immunization Programs, Primary Health Care, Nursing, Utilization, Public Health Informatics, Sistemas de Informação em Saúde, Programas de Imunização, Atenção Primária à Saúde, Enfermagem, Utilização, Informática em Saúde Pública, Sistemas de Información en Salud, Programas de Inmunización, Atención Primaria de Salud, Enfermería, Utilización, Informática en Salud Pública

## Abstract

**Objective:**

to analyze the acceptance and use of the Information System of the National Immunization Program in primary health care vaccination rooms.

**Method:**

a unique case study of a qualitative approach in the light of the Unified Theory of Acceptance and Use of Technology. Data collection included an interview with 18 professionals responsible for the implementation of the information system, observation of vaccination rooms in 12 municipalities of the West Macro-region of Minas Gerais, selected from a preliminary study. Data was systematized and analyzed through Content Analysis.

**Results:**

the interviewees are satisfied with the usefulness and ease of the system usage, but do not have the same satisfaction with the organizational infrastructure due to the lack of computers and low Internet connectivity in the health units, as well as with the incipient training for the use of the information system and the lack of skills with the technology among the human resources.

**Conclusion:**

nursing professionals perceive advantages in the acceptance and use of the Information System of the National Immunization Program. It was clear that the vaccinated individual’s history control and the decrease of records in paper are evidenced as facilitators of this acceptance. The system was considered reliable and secure.

## Abstract (pt)

**Objetivo:**

analisar a aceitação e o uso do Sistema de Informação do Programa Nacional de Imunização nas salas de vacinação da atenção primária à saúde.

**Método:**

estudo de caso único de abordagem qualitativa à luz da Teoria Unificada de Aceitação e Uso de Tecnologia. A coleta de dados compreendeu entrevista com 18 profissionais, responsáveis pela implantação do sistema de informação, observação das salas de vacinação de 12 municípios da Macrorregião Oeste de Minas Gerais, selecionados a partir de estudo preliminar. Os dados foram sistematizados e analisados por meio da Análise de Conteúdo.

**Resultados:**

os entrevistados sentem-se satisfeitos com a utilidade e facilidade de uso do sistema, porém não apresentam a mesma satisfação com a infraestrutura organizacional devido à falta de computadores e à baixa conectividade da internet nas unidades de saúde, além da incipiente capacitação para utilização do sistema de informação e da falta de habilidade dos recursos humanos com a tecnologia.

**Conclusão:**

os profissionais de enfermagem percebem vantagens na aceitação e uso do Sistema de Informação do Programa Nacional de Imunização. Revela-se que o controle do histórico do vacinado e a diminuição de registros em papéis são evidenciados como facilitadores desta aceitação. O Sistema foi considerado confiável e seguro.

## Abstract (es)

**Objetivo:**

analizar la aceptación y uso del Sistema de Información del Programa Nacional de Inmunización en centros de vacunación de atención primaria de salud.

**Método:**

estudio de caso único con un enfoque cualitativo a la luz de la Teoría Unificada de Aceptación y Uso de Tecnología. La recopilación de datos comprendió una entrevista con 18 profesionales, responsables de implementar el sistema de información, observando los centros de vacunación de 12 municipios en la Macrorregión Oeste de Minas Gerais, seleccionados en función de un estudio preliminar. Los datos fueron sistematizados y analizados utilizando el Análisis de Contenido.

**Resultados:**

los entrevistados están satisfechos con la utilidad y facilidad de uso del sistema, pero no muestran la misma satisfacción con la infraestructura organizacional debido a la falta de computadoras y a la baja conectividad a Internet en los centros de salud. A estos factores deben sumarse la capacitación incipiente para el uso del sistema de información y la falta de habilidad de los recursos humanos con la tecnología.

**Conclusión:**

los profesionales de enfermería perciben ventajas en la aceptación y uso del Sistema de Información del Programa Nacional de Inmunización. Se revela que el control del historial de vacunación y la reducción de los registros en papel se evidencian como facilitadores de esta aceptación. El sistema se consideró confiable y seguro.

## Introduction

Computerized Immunization Systems *(Sistemas Informatizados de Imunização*, SIIs) are important tools for evaluating and monitoring immunization programs, both at local and national levels, by providing up-to-date data^(^ 1 ^-^ 3 ^)^ and their use promotes equity of access to immunobiological agents and identification of low coverage pockets^(^ 4 ^)^.

In a systematic review study, conducted by the Community Preventive Services Task Force, in 108 published papers and 132 conference abstracts, it was identified that SIIs are effective in increasing vaccine coverage and, consequently, reducing vaccine-preventable diseases by means of their ability to determine client’s immunization status and support clinical decisions^(^ 5 ^)^.

For this reason, since the early 2000s, the Center for Disease Control and Prevention (CDC) has recognized the SII as an essential component of immunization programs and defines it as confidential, population-based computerized systems for maintaining information on immunization^(^ 6 ^)^.

The first SIIs, at the initiative of service providers, date back to the 1970s in the United Kingdom and the USA, and to the late 1970s in Canada^(^ 7 ^)^. In Brazil, since 2010, the National Immunization Program Information System (*Sistema de Informação do Programa Nacional de Imunização,* SIPNI) has been in the process of implementation, and it allows for the most accurate assessment of coverage and identification of the vaccinated individual^(^ 4 ^,^ 8 ^)^.

The SIPNI is a system developed by the Department of Health of the Brazilian Public Health System (*Departamento de Saúde do Sistema Único de Saúde*, DATASUS) to enable risk assessment regarding the occurrence of outbreaks and epidemics of vaccine-preventable diseases, based on the registration of immunobiological applied to a given population, aggregated by age group, time period and geographical area. It also controls the stock of immunobiological agents and indications of special immunobiologicals and their adverse events^(^ 8 ^)^. The SIPNI can be found in the desktop version and used off-line, with encrypted file submission on a website and, in the on-line version, it is already implemented in several municipalities in the state of Minas Gerais^(^ 8 ^)^.

Despite the benefits of the SIPNI, its implementation has been occurring slowly and heterogeneously in the municipalities. In 2016 the implementation process in the states and municipalities was below 60%. In the meantime, the success of the implementation depends on the acceptance and use of the system, which must be studied in order to implement an efficient and sustainable computerized system, capable of being present throughout the national territory and meeting the needs of Brazil’s complex National Immunization Program^(^ 4 ^)^.

Studies and research on acceptance of technologies, such as those on information systems, have been written in recent years under the most diverse approaches. This is justified by the significant increase in the use of these systems in various activities, which changes the relationship in all social spheres^(^ 9 ^)^. The introduction of computerized systems in the field of health contributes to the organization of services, to communication and to the improvement of the quality of the provided care. However, some factors - such as lack of knowledge and of resources and infrastructure - have influenced the acceptance and use of the system^(^ 10 ^)^. Based on these considerations, the guiding question for this study is the following: What factors have influenced the acceptance and use of the SIPNI in primary health care vaccination rooms?

For these reasons, the objective of the study is to analyze the acceptance and use of the Information System of the National Immunization Program in primary health care vaccination rooms.

## Method

This is a unique case study^(^ 11 ^)^, using the Unified Theory of Acceptance and Use of Technology (UTAUT) model with a qualitative approach. As the unit of analysis, we define the acceptance and use of the SIPNI and, as a context, the municipalities of the West Macro-region of Minas Gerais.

The UTAUT model aims to explain an individual’s intention to use a technology device and considers four key attributes in determining acceptance and use (performance expectation, effort expectation, enabling conditions and social influence) influenced by age, gender, experience, and voluntariness, as shown in Figure 1 ^(^ 9 ^)^.

**Figure 1:** Demonstration of the fundamental attributes in determining acceptance and use, based on the UTAUT model. Minas Gerais, Brazil, 2018

Attributes	Description
Performance expectation	The individual believes that using the system will help them to make gains in work performance.
Effort expectation	It concerns the ease of use of the information system.
Social influence	It consists of an individual's perception of how important it is, for close and relevant people, that they use the system.
Enabling conditions	The individual believes that the existing organizational and technical infrastructure in the company is sufficient to support the use of the information system.

Source: Adapted from Venkatesh, et al. 2003

The West Macro-region of Minas Gerais, located between the Central, Southern and Upper Paranaíba regions, which covers about 1,364,023 inhabitants and is composed of 54 municipalities, was intentionally defined as the scenario^(^ 12 ^)^. For data collection, 12 municipalities in this region were selected.

The selection of municipalities was based on a preliminary study, with a quantitative approach, conducted in the 54 municipalities, to assess the implementation degree of the SIPNI. The municipalities were categorized according to scores established in: adequate implementation (80.0 to 100%); partially adequate implementation (60.0 to >79.9%); inadequate implementation (40.0 to >59.9%); critical implementation (less than 40.0%)^(^ 13 ^)^. Four municipalities were intentionally selected out of each category of implementation degree, with the largest number of vaccination rooms. We highlight that no municipality has reached the adequate implementation category. Thus, 12 municipalities participated in the study. The number of vaccination rooms *per* municipality varied from 5 to 21 rooms, with the web version of the SIPNI present in 70.5% of the rooms.

The professionals from the 12 selected municipalities that were involved in the implementation of the SIPNI were listed as study participants. In most municipalities, those involved in implementing the system were the professionals designated to occupy the function of Technical Reference (TR) in immunization. However, in five municipalities, besides the TR in immunization, we interviewed the primary health care coordinator, nurse of the Family Health Strategy, the Municipal Secretariat of Health and a community health agent, all involved in the implementation. Besides those involved in the SIPNI implementation in the municipalities, the professional responsible for this implementation in the West Macro-region and the reference professional for the system in the Minas Gerais state were interviewed, totaling 18 professionals, who were contacted and agreed to participate in the survey.

Data collection took place from June to July 2018, with the use of an individual interview, based on a semi-structured script, addressing guiding questions regarding the attributes of the UTAUT theory (performance expectation, effort expectation, social influence, and enabling conditions). The script included characteristics that contribute to the adoption/use; the decision process for the SIPNI implementation; the process for the adoption/use of the SIPNI from the beginning; key individuals in the process of the SIPNI adoption/use; information sources for access to the SIPNI, and analysis regarding the system used previously. The interviews took place in the professional’s own workplace and were recorded, transcribed in full and lasted an average of 15 minutes. In each municipality a vaccination room was selected for a technical visit in order to learn about the type of SIPNI used, Internet connectivity, to identify the limitations in the use of the technology, when and who was for typing into the SIPNI, and to learn how the process of implementing the SIPNI and the training to use the system was performed. The data from the technical visits were recorded in memos, generating the Observation Notes (ONs), as well as the impressions of the researcher during the interviews. In order to maintain the anonymity of the research participants, the interviewees were coded with the letter E followed by the chronological sequence of the interviews.

The data were analyzed using the Content Analysis technique, Thematic-Categorical Mode^(^ 14 ^)^. For the application of the content analysis, the stages of pre-analysis, material exploration, or codification and treatment of the results obtained by interpretation were used. In the pre-analysis, or floating reading of the interviews, the key points were identified, that is, the main points addressed by the interviewees in each question from the script. The material was then exploited and coded. The registration units, meaning units, context units, and thematic categories were extracted. The categories can be created *a priori* or *a posteriori*. When defined *a priori,* validity or relevance can be built from a theoretical base^(^ 14 ^)^. So, the categories were defined *a priori*, according to the four UTAUT model attributes: performance expectation, effort expectation, enabling conditions and social influence. In the last phase, the results were treated in such a way as to be significant and valid for the elaboration of the emerging research model, based on the Unified Theory of Acceptance and Use of Technology, from the interviews, and the ONs, empirical data, and theoretical references, seeking to answer the research’s guiding question.

This research, in which the guidelines of the National Health Council were followed, began after the approval of the Ethics Committee for Research on Human Beings of the Ribeirão Preto School of Nursing of the University of São Paulo (*Escola de Enfermagem de Ribeirão Preto da Universidade de São Paulo*, EERP/USP), under Opinion No. 2,768,82.

## Results

We interviewed thirteen nurses, one community health worker, two nursing technicians, one biomedical, and one public manager. Age ranged from 27 to 57 years old, with a mean of 38 years. There was predominance of females (94.4%). Also, six interviewees had specialization of the *lato sensu* type and two were masters. The length of service in primary health care ranged from 1 to 32 years, most of them served in it for more than 10 years. Four interviewees were hired on a contract basis, while the others were approved in a public contest.

**Figure 2:**
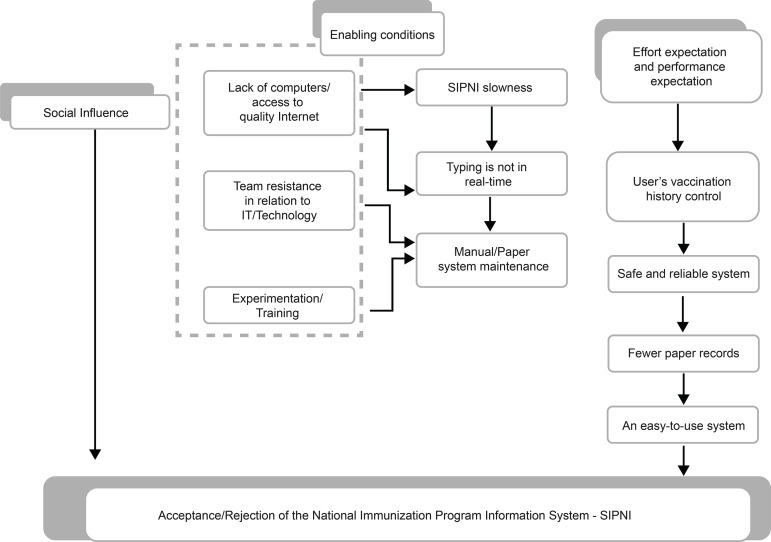
A theoretical model of acceptance/use of the Information System of the National Immunization Program. West Macro-region of Minas Gerais, Brazil, 2018

From the process of data analysis and integration emerged the theoretical model of the research, supported by categories based on the four attributes of the UTAUT theory, as illustrated in Figure 2.

### Performance expectation

In the analytical category performance expectation, the respondents considered some advantages in using the SIPNI: *The system* (SIPNI) *is theoretically great. Because, then, the data are filed and we can get access to them in another city, if the child gets vaccinated in another city, I will know it from here* (E1). *The duplicity of vaccines can be avoided, as well as avoid having to repeat the scheme if the individual says they have taken it, but since they have no proof, we cannot consider* (E15); *What has contributed, which I thought was of great value, was this issue of coverage, not getting data lost, and of assessing coverage, which makes it much easier to get the reports* (E4); *Actually, I think that the* SIPNI *was an innovation for us. I, in particular, think it’s the best thing they’ve done in many years* (E7).

The participants presented comparisons between the paper system used previously and technological innovation. They verbalized that the SIPNI brought improvements in the daily work in the vaccination room: *It made it a lot easier. Before, I used to spend about five sheet papers because I put it on the child’s card, on the mirror card, on my records, at the end of the service, on another paper and the active search* (E3); *Any information you need is much quicker to search the program than the notebook, you understand? I think it helps a lot* (E5); *I think it is great. In the old days, we used paper* (E10); *But then, looking back, whoever has ever worked on the tip* (Primary Health Care Unit), *using the* SIPNI *is fantastic. For me, it is a treasure* (E14).

Other perceived advantages were the minimization of errors in the vaccination room and confidence in the new system: *And also, if used correctly, it greatly minimizes errors in the vaccine room* (E7); *Now much better, more reliable* (E8).

### Effort expectation

In the effort expectation, the respondents verbalized that they find the system easy to handle: *The SIPNI is easy to handle, it is not those difficult programs* (E2); *Anyone with a login and password, logs in and accesses it. Works with it normally. It offers no difficulty. It is a really good, resolute program with a simplified language* (E6); *Sometimes the obstacles are the people themselves who have trouble dealing with computers. They graduated a long time ago* (E10).

However, the lack of skills of the human resources with the technology was highlighted, especially older people, who cannot use the system, some do not operate the computer: *Here in this unit, in particular, we have three technicians* (nursing technicians), *all three are old, almost retiring. So, they have too much resistance to the system, because they have no access to the Internet, and are not familiar with computers* (E1); *We see that people who have problems with computers have a problem with the* SIPNI (E12); *The hardest part was with the technicians, for them to learn how to use it, even turning on the computer. Some of them did not know even turn on the computer* (E16); *Older people have difficulty, some do not even touch it* (E2); *You have a lot of professionals who have trouble even messing with the computer. There are rooms wherein the technician does not type, just the nurse* (E18).

### Enabling conditions

The Enabling Conditions analytical category discusses the organizational and technical infrastructure needed to use the information system. The study participants reported that the optimal quantity of computers and a good quality Internet are essential for the use of the system: *But what impairs the* SIPNI *in the units is the Internet connection. It’s the Internet of poor quality, as for other things, there is no problem messing with it* (E6); *We have only one computer in the unit, the one in the vaccine room. So, for all that you have to solve, it is only a computer for everyone. And there is also the Internet issue, as the Ministry launched a program that depends on the Internet, but did not give support to the units* (E1); *The difficulty that we have here at our* FHP (Family Health Program), *is the Internet connection, to use it, for it only keeps on loading. Sometimes you need it more desperately* (E3); *During a visit to a vaccine room, low internet connectivity was identified and the nursing technician records everything in the notebook and types the reference in immunization in the Municipal Health Secretariat office* (ON).

The lack of good quality Internet leads to the slowness of the system, as recalled by some interviewees and, consequently, in some municipalities to the maintenance of paper records. This negative influence, generated by innovation, on the assistance in the vaccination room, overloads the professional who needs to register on paper and then double work by typing it into the system: *Because there is no time. It sometimes freezes on a single screen for a long time and doesn’t conclude loading. Then we end up having to do it on the notebook, on the mirror card, on the child’s card and from that notebook we pass it to the* SIPNI (E11); *We have a problem with the Internet connection, there are units where the Internet is not very good. So we have to type it later* (E12); *In a unit with a web system, the CHAs (Community Health Agents) are the ones who feed the* SIPNI. *There is a scale for the CHAs typing it into the* SIPNI. *It is not in real time* (ON).

Keeping a duplicate paper record affects daily work and has an effect on vaccination coverage targets: *Because then our vaccination coverage targets get down the index, for we can’t type it into the system in time. Actually, there is no unvaccinated kid but there are records not entered into the system* (E1); *The* SIPNI *has come precisely to help us in this issue of not reaching the goal of vaccination coverage. But, what happened? It got a lot worse* (E7); *We never reach the goal, and then we go to the practice, in everyday life, we look at the mirror card and it is complete, the children are vaccinated. We have this gap that we can’t solve, what is in the system is not what is in practice* (E9).

The system has been implemented in a sudden, non-systematized way and without training for the workers: *As I reported, when the* SIPNI *was deployed, it was a very quick thing, deployed overnight and we had to use it. So, now, we are deploying the* SIPNI, *we have to start using the* SIPNI, *and it was like this, overnight. Nobody’s had a chance to talk like that: “Let’s get ready before deploying it”* (E7); *So it was kind of out of the blue, we didn’t have the training. I learned the SIPNI out of curiosity* (E6); [...] *Give help, offer training until the person memorizes it, that this is important. So I think that is something that would make it easier and help too. We did not have that* (E7).

The training sessions were held by the municipal, regional, and state health secretariats; however, this process was not always resolute: *The trained person does not understand vaccines and does not work on typing. So they’re going to have a hard time passing it on.* [...] *there must have a little knowledge of the vaccine and multiplier feature* (E17); *And, unfortunately, I was unable to attend the municipalities* [...]. *It is only one person, one professional in the Municipal Health Secretariat. Now the* SIPNI *is in each room. So, what I could solve was from here, often over the phone, me standing here, and he there, leading the process* (E14).

However, in the municipalities that carried out the training, a minimization of resistance to the new system was evidenced: *But the training allowed that to be diminished, you know?! So they could understand the importance of technological evolution, the qualification of the service* (E9); [...] *There was the training there at the Regional* (Regional Health Secretariat), *and then I passed it on to the rest of the team. And so this has contributed a great deal* (E2).

### Social influence

Another item evaluated was the social influence of the system on the acceptance of the innovation. The imposition of higher management spheres influences the decision to use the innovation and makes an individual simply change their intention in response to social pressure: *Today we already know it is something that is part of our routine. Unfortunately, it is something we have to adapt* (E12); *This system has to be used. The municipality has to abide by* (E10); *But what we put in for everyone is that we all needed to learn* (E15); *Now 100% of the state is using the* SIPNI, *or the system of their own, which they integrate into the* SIPNI (E15).

## Discussion

In the analysis of acceptance and use of the SIPNI, aspects that enhance its acceptance were highlighted. The respondents are satisfied with the usefulness and the easy use of the system but do not have the same satisfaction with the organizational infrastructure due to the lack of computers, the low Internet connectivity in health facilities, the incipient training in the use of the information system, and the lack of skills of the some human resources with the technology.

In the UTAUT theory, acceptance of a new system is related to users’ perception that the value gained from adopting it can be greater than the challenges and effort spent learning how to handle it and changing the previous way of doing things^(^ 3 ^)^. In the context of the SIPNI, in the speeches, the advantage was evidenced of using it instead of the manual record, previously used, which consumed much of the time with records in several roles, besides increasing the fragmentation of the vaccination records. Another recognized benefit, with the adoption of the system, was the ability to easily access and retrieve data from the patient’s vaccination history in different Brazilian municipalities integrated online. This reduces the administration of unnecessary doses, increasing safety by avoiding adverse events, and costs for the SIPNI.

The implementation of an information system is a complex and multidimensional process influenced by technical, individual, human, and organizational factors which must be approached innovatively, according to the specific needs of each system and each group of users^(^ 15 ^)^. In the context of primary care, the precariousness of material inputs required for the use of information systems, such as technological devices and the Internet, negatively affects the process of acceptance and use of information systems^(^ 16 ^-^ 17 ^)^.

One of the limiting factors, identified in most municipalities with a web system, was the slow Internet connectivity, leading to the slowness of the information system and, consequently, to the maintenance of the paper record and to the non-input of data in real-time. This result corroborates the findings of a study conducted in Kenya to evaluate an electronic health information system with an immunization component, which identified barriers such as power outage, slow Internet connectivity, the time required for data entry, paper, and system data entry^(^ 18 ^)^.

If the SII is not properly fed with real-time data entry, it may generate under-registration, compromising the integration of nominal vaccination data and, consequently, the range of vaccine coverage^(^ 7 ^)^. The time between vaccination and the data entered into the SII must be minimized so that the information comes to be in real-time^(^ 19 ^)^.

According to the UTAUT theory model, the acceptance of technology is related to the individual’s belief in the contribution of the technological resource use to the improvement of the quality of their work^(^ 9 ^)^. During the technical visit in one municipality we identified that, despite the availability of the system via web in all rooms, the operation of the entire work process in the vaccination room is done manually, with the paper registration. In the studied context, the team registers in the mirror card, in the patient’s medical chart, in the vaccination diary control and does not enter the vaccine in real-time. Each unit has a system feeding schedule. The professionals complained a lot about the time spent to do everything manually and then enter it in the system. Several studies indicate that the intention to use information systems is related to the perception of their applicability in problem-solving and decision making^(^ 20 ^-^ 21 ^)^.

In this sense, the incipient use before the simultaneous use of other forms of paper registration, to the detriment of the SIPNI, may hinder the perception of the potential of this system for the performance of the service. A similar phenomenon was observed in the context of the e-SUS Basic Care (e-SUS AB) strategy, in which paper-based registration and the information system emerged as an important incompatibility between the use of technology and traditional means of registration^(^ 16 ^)^. In the interviews, the participants highlighted influences of this whole process of not typing in real-time on the reach of vaccination coverage. Vaccine coverage is considered a monitoring pillar of the SII^(^ 1 ^)^. The still immature use of the SIPNI reveals the fragility of the data produced from the system. For the interviewees, the information in the system does not reflect the actual scenario of vaccination coverage, which consequently may lead to unsafe use of the information by professionals, in addition to allowing data underreporting.

The discrepancy of the SIPNI data was pointed out in a study when it describes the risk classification of vaccine-preventable diseases in Brazilian municipalities. According to the authors, most municipalities presented a high risk of disease; their findings pointed to possible inconsistencies in the SIPNI data capable of distorting vaccination coverage and abandonment proportions, interfering with the risk indicator^(^ 22 ^)^.

In implementing changes that affect the structure, culture, work processes, behavior, and communication channels of a health care organization, some resistance is expected^(^ 23 ^)^. One solution is to conduct progressive training and educational activities^(^ 17 ^)^. In a systematic review it was identified in the studies that, where there was adequate technical support and training, the acceptance of the information system was easier. In contrast, in studies in which inadequate or non-existent IT support or training was reported, the tendency was to conclude that these factors were barriers to system implementation^(^ 15 ^)^. However, as well as the implementation of e-SUS AB in the scenario studied, the SIPNI has also been implemented in a sudden and vertical way^(^ 16 ^)^, without an offer (or with an insufficient offer) of training, which may compromise its acceptance.

In implementing an information system in a big teaching hospital in the UK, it was identified that the way people reacted to the adoption and implementation of the system was influenced by age and attitude towards Information and Communication Technologies (ICTs). Younger employees, who were familiar with computers, easily accepted the technology, unlike older physicians and nurses, who were reluctant to do so^(^ 23 ^)^.

The findings of this study indicate that older nursing professionals and/or those with little familiarity with technology tend to be more resistant to the SIPNI innovation. According to the UTAUT theory, the ease of the information system usage is influenced by age, in such a way that the effect will be stronger for older workers, with increasing experience^(^ 9 ^)^. The acceptance and use of electronic recording by nurses was the subject of a literature review, in which the nurses’ low acceptance of the technology was related to the lack of perception on advantages of its use due to incipient use^(^ 24 ^)^. Another aspect listed by the authors was the effects of the characteristics of the technology itself, but mainly the personal specificities of the nurses affecting greatly the acceptance of technological artifacts. In this sense, organizational psychologists have realized that older workers, due to the increase in cognitive and physical limitations associated with age, assign greater importance to receiving help in the context of using information technologies^(^ 9 ^)^. However, even in the face of resistance to the use of information systems, technology has become an indispensable tool in the health scope. Studies show that preparation/training in the use of systems was related to ease of use as it improves people’s skills with technology, favoring the perception of its usefulness and ease of handling it^(^ 15 ^,^ 17 ^)^. Social pressure in the use of innovation tends to attenuate over time, as experience increases, and in the UTAUT theory it is suggested that women tend to be more sensitive to the opinions of others and, therefore, social influence becomes more relevant in the intention to use the new technology^(^ 9 ^)^.

In Brazil, the predominantly female nursing team^(^ 25 ^)^ is responsible for immunization activities. Considering this data, the acceptance of the SIPNI, identified in this study, may be influenced by this female predominance in the vaccination room. In this sense, it is important in health courses to prepare students for the use of technological resources, aiming at training in the use of information technology as an ally of care^(^ 17 ^)^, considering the context of growing dependence on information technology.

Innovation alone is not enough to impact on the quality of the services provided. It is essential that service planners and managers understand the human and organizational processes involved in motivating change and adopting innovation^(^ 23 ^)^.

This study, of a qualitative nature, has the limitation of not statistically demonstrating the relationships and power of each of the attributes in the acceptance and use of the SIPNI, besides the lack of data generalization, because it is a case study in a macro-region. However, the unveiling of the difficulties faced in the process of acceptance and use of the SIPNI in the macro-region is presented as a strength.

The results of the research bring contributions to the implementation of an information system that really enables complete and correct vaccination records, with electronic access in real-time. Also, it is important to highlight the unprecedented nature of the study, as this is a recent innovation in the vaccination room, and the scarcity of studies with the theoretical approach used.

## Conclusion

Nursing professionals perceive advantages in the acceptance and use of the SIPNI. It was revealed that the control of the vaccination history and the reduction in paper records were evidenced as facilitators of this acceptance. Furthermore, the SIPNI was considered reliable and safe.

It is important that new research is triggered to quantitatively analyze the influence of each attribute of the UTAUT theory on the SIPNI acceptance and use.
